# Standardization of laparoscopic intraperitoneal onlay mesh repair for incisional hernia: Impact on clinical outcome and quality-of-life (LIPOM trial, NCT 02089958)

**DOI:** 10.1016/j.conctc.2025.101481

**Published:** 2025-03-29

**Authors:** A. Hellinger, F. Wotzlaw, V. Fackeldey, G. Pistorius, M. Zdichavsky, O. Stern, R. Jünemann, A. Buia, LIPOM trial Study Group

**Affiliations:** a Department of General, Visceral, Endocrine and Oncologic Surgery, Universitätsmedizin Marburg Campus Fulda, Klinikum Fulda, Germany; b Department of General, Vascular and Visceral Surgery, Klinik Kitzinger Land, Kitzingen, Germany; c Department of General, Thoracic and Visceral Surgery, Sozialstiftung Bamberg, Bamberg, Germany; d Department of General, Visceral, Thoracic, and Trauma Surgery, Filderklinik, Filderstadt-Bonlanden, Germany; e Department of General and Visceral Surgery, Asklepios Klinik Wandsbeck, Hamburg, Germany; f StatConsult, Gesellschaft für klinische und Versorgungsforschung mbH, Magdeburg, Germany; g Department of General and Thoracic Surgery, Asklepios Klinik, Langen, Germany

**Keywords:** Incisional hernia, Physiomesh™, Laparoscopic intraperitoneal onlay mesh augmentation, Standardization, Clinical outcome, Patient reported outcome

## Abstract

**Purpose:**

Current available outcome data following laparoscopic intraperitoneal onlay mesh repair (IPOM) for incisional hernia (IH) are comparable to a limited extent only because of a huge number of variability particulary in surgical technique and use of medical devices. In this prospective observational multicenter cohort study we evaluate the impact of a consensus driven standard protocol for IPOM, that is mesh fixation with absorbable tacks in double crown technique enforced by additional non-absorbable transfascial sutures at the edges of the mesh along with the use of Physiomesh™, on clinical and patient reported outcome measures.

**Methods:**

A total of 102 consecutive patients were screened for eligibility between September 2013 and October 2014. 85 patients (IH: EHS W1: n = 39, W2: n = 46) were included into the study. Clinical examination and PROM for pain and quality of life measure (Carolina Comfort Scale, CCS) were performed at baseline, during hospital stay and at 6 weeks, 6 months and 1 year follow-up.

**Results:**

The follow-up rate was 87,1 % for the primary endpoint. The Kaplan-Meier estimate for freedom-of-recurrence at 1 year was 95.9 % (95 %-CI: 87.9–98.7 %), the cumulative recurrence rate at 1 year was 4.1 % (95 %-CI: 0.9–11.7 %). There was no intraoperative complication. One seroma (type I; 1/84 (1,2 %), 95 %-CI: 0–7.1 %) was diagnosed during hospital stay. 12 seroma (n = 12/74 (16,2 %), 95 %-CI: 9.4–26.4 %; n = 10 type II and n = 2 type IV) were diagnosed during follow-up requiring surgical intervention in 1 patient (1,4 %, Clavien Dindo grade IIIb). Subcutaneous hematoma were found during follow-up in 8 patients (8/75 (10.7 %); 95 %-CI: 5.3–19.9) with need for surgical intervention in 2 patients (2,7 %, Clavien Dindo grade IIIb). There were 3 superficial surgical site infections (3/74 (4,1 %); 95 %-CI: 0.9–11.7 %, Clavien-Dindo grade I) without need for reoperation. Patient reported pain as measured by numeric rating scale at baseline was 1.36 ± 1.53 and 0.35 ± 1.04 at 1 year follow-up. CCS total scores indicated a good outcome with a decrease to 2.80 ± 6.47 (Min: 0, Q1: 0, Median: 0; Q3: 3.0; Max: 38.0) at 1 year follow-up. Subscore sensation of mesh decreased from 4.01 ± 6.76 (min: 0, Q1: 0, Median: 0; Q3: 5.0; Max: 28.0) at 6 weeks to 1.67 ± 3.99 (Min: 0, Q1: 0, Median: 0; Q3: 1.0; Max: 21.0) after 6 months and 0.90 ± 2.69 (Min: 0, Q1: 0, Median: 0; Q3: 0; Max: 14.0) after 1 year follow-up. Subscores pain and movement limitation both decreased during follow-up and were significantly reduced at 1 year compared to preoperative assessment (p < 0.001).

**Conclusions:**

This study showed favourable clinical and patient reported outcomes and a low rate of recurrences at 1 year follow-up after IPOM for elective IH applying a standardized surgical technique including the use of Physiomesh™. In view of the data leading to the market withdrawal of the Physiomesh™, one might conclude, that the described standard may have contributed to a compensation of the suspected delay in tissue integration of the Physiomesh™ resulting in a more proper mesh fixation compared to absorbable tack fixation with/without absorbable TFS. This may lead to the general appraisal, that the fixation technique has to be adapted to the individual characteristics of type of mesh and fixation devices and the planned anatomic landing zone of the mesh.

## 1 Introduction

Laparoscopic intraperitoneal onlay mesh repair (IPOM) for incisional hernia (IH) basically subsumes tensionless bridging of the hernia gap with a composite mesh (Standard IPOM; sIPOM) and the advancement of this technique with gap closure and composite mesh augmentation in order to restore functional integrity of the abdominal wall (suturing concept; IPOM-plus), [[1], [2], [3], [4], [5]]. Both principles of IPOM are widely disseminated and are common part of an individual, tailored approach in the treatment of IH by using different open, minimally invasive or both (hybrid) surgical techniques [6,7]. However, IPOM is far away to reach a standard due to a huge number of variations i. e. patient's selection, type of hernia, defect size, use of type of mesh, use of type of fixation device, surgical technique with focus on mesh overlap/size and concepts of fixation. Besides this heterogeneity, current available data and evidence are further limited because of immanent surgical and methodical variations i. e. learning curve and selection bias caused by analyzing IH and ventral hernia as one entity [8,9]. This is in line with the results of an expert questionnaire published by Pawlak, and current available clinical guidelines and consensus statements [[10], [11], [12], [13], [14], [15], [16]]. All attempts to provide further evidence outline the above mentioned variability almost resulting in recommendations of low to moderate strength or at least expert opinions in terms of clinical practice guidelines to treat patients suffering from IH with the best possible care and to ensure best patient safety [17]. In order to evaluate the impact of a reduction of the described heterogeneity of IPOM for IH on clinical outcome measures, we developed a standard protocol for IPOM based on a consensus driven process (LIPOM trial).

## 2 Methods

### 2.1 Trial design

The LIPOM (Standardization of laparoscopic intraperitoneal onlay mesh repair) trial was designed as a non-comparative, open prospective multicenter observational cohort study according to the recommendations for reporting outcome results in abdominal wall repair (Palermo consensus meeting), the STROBE Initiative (Strengthening the Reporting of Observational Studies in Epidemiology) and the IDEAL (Idea, Development, Exploration, Assessment, Long-term Follow-up, Improving the Quality of Research in Surgery) Framework for Surgical Innovation (stage 2b, exploration) [[18], [19], [20]]. The study design in detail has been previously published [21]. In brief, health care outcome measures were assessed in a consecutive cohort of patients with symptomatic incisional hernias treated by IPOM. The latter was based on a standard protocol as developed by a consensus process according to the proposals from the National Institute of Health. Participants were five board-certified surgeons (members of the Hernia panel (HP), see Appendix) with experience and readiness in IPOM. Physiomesh™ and Securestrap™ (Johnson&Johnson Medical GmbH, Norderstedt, Germany) were identified as medical devices for standard use in the trial based on existing evidence at planning the study in May 2013 [[22], [23], [24], [25]]. In order to minimize bias due to institutional and surgical experience as well as deviation from the standard protocol, the following aspects have to be fulfilled: personal and institutional case-load of more than 20 procedures per year, board certification of partizipating surgeons, informed consent for the standard protocol, acceptance of external monitoring by members of the HP with a visitation before beginning and at the end of the study, documentation of procedural steps by digital imaging of each procedure (after adhesiolysis, measurement of hernia size, following mesh fixation) combined with an uncut video, and a data source verification with 100 % electronic monitoring during the study.

### 2.2 Standard protocol

Patients were placed in the supine position. Three trocars were introduced into the intraabdominal cavity on the anterior axillary line or more laterally on the left or right side depending on the hernia site. A capnoperitoneum up to 20 mmHg depending on cardiac and respiratory function was established. Adhesiolysis (caution: ideally no use of devices based on the application of thermic energy) and preparation of the planned landing zone of the mesh with removement of fatty tissue and, if necessary, the spatium recii and/or the Lig. teres hepatis was completed to insure a mesh overlap of 5 cm. A resection of the hernia sac was recommended. Intraabdominal measurement of the defect size was performed under low pressure capnoperitoneum (≤8 mmHg) in accordance with the EHS guidelines [26]. The mesh size was calculated by adding up the defect size and the planned overlap of 5 cm in both, sIPOM and IPOM-plus. The Physiomesh™, which is a lightweight polypropylene intraperitoneal onlay mesh coated on both, the visceral and parietal surfaces with a poliglecaprone-25 anti-adhesive absorbable layer encapsulated with polydioxanone, was configurated backtable. Furterhmore, it was armed with a nonabsorbable suture (polypropylene) each at the edges of the mesh for transfascial suture (TFS) fixation of mesh after implantation. In IPOM-plus, gap closure was performed with nonabsorbable sutures according to the suturing concept as described by Chelala [2]. Following intraabdominal positioning of the mesh, fixation was performed under low pressure capnoperitoneum (≤8 mmHg) to ensure optimal contact at the prosthetic-peritoneal interface as follows: the mesh was transfascially fixed with the 4 nonabsorbable sutures at the edges of the mesh after retrieving all threads in front of the abdominal wall through small skin incisions. Mesh fixation was completed due to circumferentially anchoring to the abdominal wall with the SecureStrap™, which is an absorbable strap fixation device proposed for fixation of the Physiomesh™, in double crown technique (outer line: distance to the edges 0,5 cm, distance between tackers 2 cm, inner line: distance 1 cm to the gap edge, distance between tackers 2 cm). Trocar incisions with a size more than 5 mm are closed. Postoperative analgesia was performed according to the WHO scheme based on patient reported pain using the numeric rating scale (NRS).

### 2.3 Inclusion and exclusion criteria

Patients were included if they met the following criteria: (i) age ≥18 years; (ii) primary incisional hernia; (iii) symptomatic/progressive hernia; (iv) hernia size <10 cm (EHS W 1–2); (v) hernia location according to EHS of M1-5 and L1-3; and (vi) written informed consent for partizipation. Patients were excluded if they met any of the following criteria: (i) recurrent incisional hernia; (ii) primary ventral hernia; (iii) hernia size ≥ 10 cm (EHS W3); (iv) hernia location classified as EHS L4; (v) simultaneous surgical intervention, e.g. appendectomy; (vi) mesh overlap <5 cm; (vii) ASA score >3; (viii) malignant disease; (ix) liver cirrhosis; (x) peritoneal carcinosis; and (xi) intraoperative lesion of large bowel.

### 2.4 Data management and monitoring

Data were documented via web access in the database “LIPOM” pursuant to the definition of the Agency of Healthcare Research and Quality (AHRQ) using an eCRF in ClinWise® Version 1.0 with automatic pseudonymization of the patient's personal data (ClinWise Health Care, StatConsult, Germany). Access to the database was limited to authentification via user name and password with free access limited to personal records. The database featured a reminder function regarding the survey of follow-up data and automatically generates correspondence to the patient. Data management, analysis of follow-up questionnaires and statistical analysis were done by StatConsult. Data safety was guaranteed by StatConsult. ClinWise® was connected via an interface to the Herniamed registry. The following adverse events were monitored: (i) wound infection, (ii) intraabdominal abscess, (iii) mesh infection, (iv) sepsis, (v) intestinal or organ injury, (vi) postoperative bleeding, (vii) pneumonia, (viii) urinary tract infection, (ix) deep vein thrombosis, (x) bowel obstruction, (xi) recurrent hernia, (xii) vomiting, and (xiii) pain at rest. Severe adverse events including (i) reoperation, (ii) ICU admission, (iii) acute incarceration, (iv) rehospitalization, and (v) death were reported to the principal investigator within 3 days. An independent Data Safety Management Board (DSMB) consisting of two non-participating experts in hernia treatment (Bernd Stechemesser MD, PAN Klinik, Köln, Germany, and Andreas Koch MD FACS, Chirurgische Praxis, Cottbus, Germany) reviews the processed data to monitor patient safety and to perform risk-benefit analysis.

### 2.5 Trial outcomes and follow-up evaluation

Patient demographic characteristics and relevant preexisting symptoms with respect to outcome measures were recorded at the screening visit. Follow-up was performed during hospital stay and after discharge at 6 weeks, 6 months and 1 year by clinical examination and patient reported outcome measurements. The primary endpoint was the 1 year recurrence rate as a time-to-event analysis for freedom-of-recurrence as determined by physical examination and ultrasound, or in case of diagnostic failure of these techniques by MRI or CT-scan. Secondary endpoints were (i) perioperative complications including bleeding, hematoma, seroma, wound infection classified according to the definition of Surgical Site Infection as described by the Centers of Disease Control and Prevention, mesh infection, bowel injury, bowel fistula, and reoperation; (ii) patient reported pain (measured by Numeric Rating Scale, NRS); (iii) patient reported health adapted quality-of-life (QoL) measured by California Comfort Scale (CCS); and (iv) mortality [18,[27], [28], [29], [30], [31]].

### 2.6 Statistical analysis

The recurrence rate has been calculated with the Kaplan-Meier estimate and the related confidence interval (time-to-event analysis) [32]. Furthermore, the cumulative recurrence rate and the related Agresti-Coull confidence interval are presented. Data are right-censored. Recurrences were collected with year and month of occurrence. For the analysis a conservative approach was applied assuming the first day of the documented month as the timepoint of recurrence. For the secondary endpoint perioperative complications with related Agresti-Coull confidence intervals are reported. Further descriptive statistics are reported for (quasi-) continuous variables (mean, standard deviation, minimum, Q1, median, Q3, maximum) and categorical variables (absolute and relative frequencies). Box-Whisker-Plots, bar charts and error bar charts are presented for patient reported pain (11-point NRS from 0 = “no pain“ to 10 = „pain as bad as you can imagine“) and QoL assessed by Carolina Comfort Scale (CCS). The CCS-questionnaire, translated into German, contains 23 items to evaluate the subscores mesh sensation (range: 0–40), pain (range: 0–40) and movement limitations (range: 0–35) resulting in a total score from 0 to 115 (CCS score), with a low score indicating a favourable QoL assessment. For preoperative evaluation, mesh related questions were excluded [30,31]. For the subscores pain and movement limitations, changes by follow-up compared to preoperative evaluation are shown in an error bar chart including mean and related confidence interval using Student's t-distribution. P-values of paired *t*-test for change of follow-up against preoperative assessment are presented. Analyses have been conducted with SAS Software version 9.4® (SAS Institute Inc., Cary, NY, USA).

## 3 Results

**Fig. 1:**
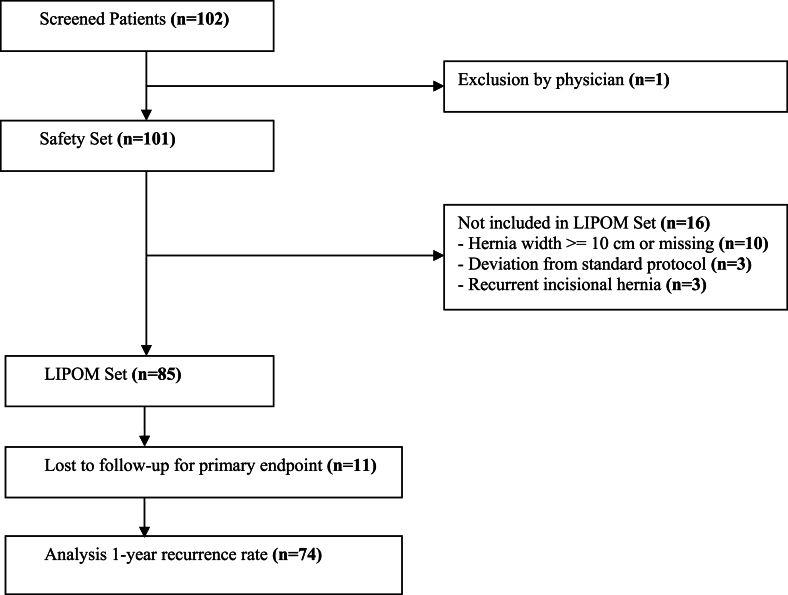
Flow diagram of the LIPOM trial.

Between September 1, 2013 and October 31, 2014 a total number of 102 consecutive patients (safety set) were screened for eligibility in 11 participating study centers (see Appendix). The flow chart for the study cohort is shown in Fig. 1. Out of the safety set, there was one patient excluded by the physician due to medical reasons. 16 patients were referred due to hernia size more than 10 cm width (EHS W3: n = 10), recurrent IH (n = 3), and deviation from standard protocol (n = 3). 85 patients were included into the LIPOM-Set with a total of 11 patients (12,9 %) lost for follow-up for the primary endpoint resulting in 74 patients (87,1 %) for analysis. Patient data at baseline and clinical characteristics are reported in Table 1.

**Table 1:** Patient data at baseline, operative data, and clinical characteristics.

Gender	Male	47 (55.3 %)
	Female	38 (44.7 %)
	Total	85 (100 %)
Age [Years]		N = 85, Mean = 59.7, SD = 12.7, Min = 25, Q1 = 53.0, Med = 60.0, Q3 = 69.0, Max = 93
BMI [kg/m^2^]		N = 85, Mean = 29.8, SD = 5.6, Min = 18.0, Q1 = 26.1, Med = 29.1, Q3 = 32.5, Max = 46.0
ASA Score	I	12 (14.1 %)
	II	46 (54.1 %)
	III	27 (31.8 %)
	IV	0 (0 %)
	Total	85 (100 %)
Duration of operation [min]		N = 85, Mean = 77.5, SD = 29.6, Min = 25, Q1 = 58.0, Med = 69.0, Q3 = 88.0, Max = 175.0
Duration of postoperative hospitalization [days]		N = 74, N(Missing) = 11, Mean = 4.6, SD = 2.4, Min = 1, Q1 = 3.0, Med = 4.0, Q3 = 5.0, Max = 14
EHS classification		
Medial/Lateral	Medial	71 (83.5 %)
	Medial + lateral	11 (12.9 %)
	Lateral	2 (2.4 %)
	Missing	1 (1.2 %)
	Total	85 (100 %)
Width	W1 (<4 cm)	39 (45.9 %)
	W2 ( ≥ 4–10 cm)	46 (54.1 %)
	Total	85 (100 %)
Gap closure	Yes	9 (10.6 %)
	No	76 (89.4 %)
	Total	85 (100 %)

N: sample size; Mean: Arithmetic Mean; SD: Empirical Standard Deviation; Min: Minimum; Q1: Lower Quartile; Med: Median; Q3: Upper Quartile; Max: Maximum; LCL: Lower Confidence Limit; UCL: Upper Confidence Limit; BMI: Body Mass Index; ASA Score: American Society of Anesthesiologists risk classification.

### 3.1 Primary endpoint

**Fig. 2:**
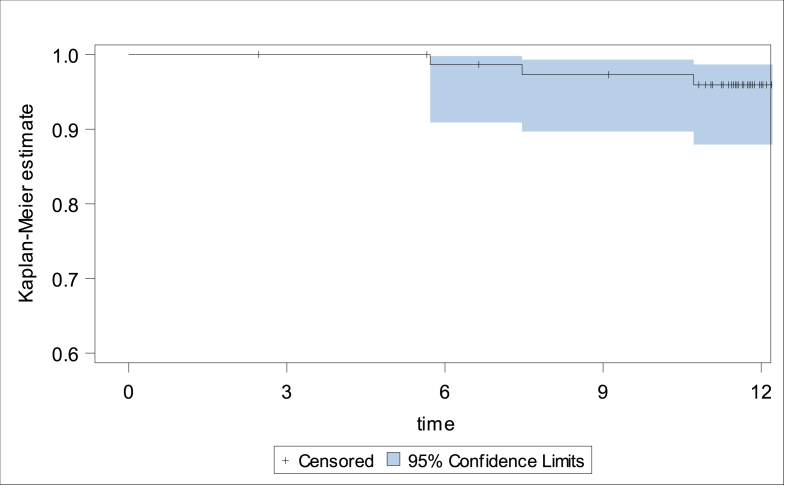
Kaplan-Meier estimate for freedom-of-recurrence.

The Kaplan-Meier estimate for freedom-of-recurrence at 1 year is 95.9 % (95 %-CI: 87.9–98.7 %), Fig. 2. During follow-up, a recurrent IH was diagnosed in 1 out of 76 patients at 6 months and 3 out of 74 patients at 1 year follow-up resulting in a cumulative recurrence rate at 1 year of 4.1 % (95 %-CI: 0.9–11.7 %).

### 3.2 Secondary endpoints

***Perioperative complications.*** No intraoperative complication was noted (n = 0/85; 95 %-CI: 0–5.2 %). During the hospital stay, there was 1 seroma (type I) diagnosed (n = 1/84 (1,2 %); 95 %-CI: 0–7.1 %), which did not require surgical intervention (reoperation n = 0 (0 %); 95 %-CI: 0–5.2 %). During follow-up, there were 8 subcutaneous hematomas (n = 8/75 (10,7 %); 95 %-CI: 5.3–19.9) and 12 seromas (n = 12/74 (16.2 %); 95 %-CI: 9.4–26.4 %; type II/IV: 11/1, respectively) diagnosed requiring surgical intervention in 2 (2,7 %; Clavien-Dindo grade IIIb) and 1 (1,4 %; Clavien-Dindo grade IIIb), respectively. 3 superficial surgical site infections (n = 3/74 (4.1 %); 95 %-CI: 0.9–11.7 %, Clavien-Dindo grade I) were treated without need for reoperation.

**Fig. 3:**
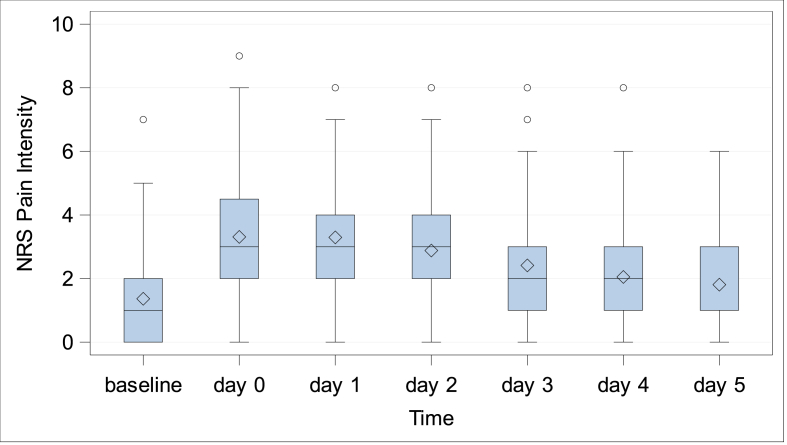
Patient reported pain intensity at preoperative screening and during postoperative follow-up in hospital.

**Fig. 4:**
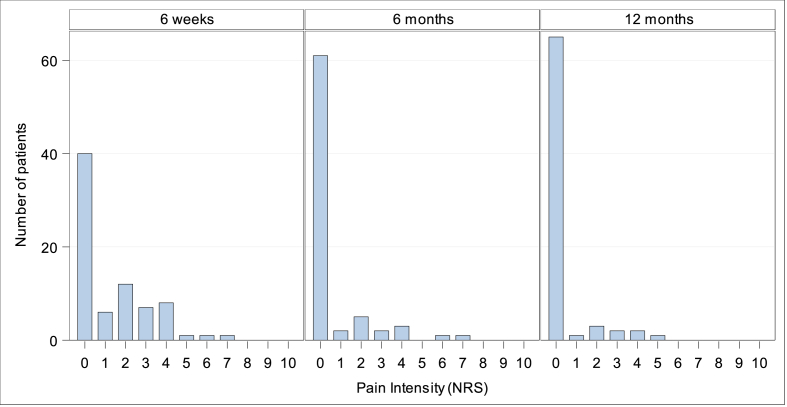
Patient-reported pain intensity during follow-up after 6 weeks, 6 month, and 1-year.

***Pain.*** Patient reported pain measured by NRS at preoperative screening was 1.36 ± 1.53 (Min: 0, Q1: 0, Median: 1.0; Q3: 2.0; Max: 7). At discharge (duration of postoperative hospitalization: 4.6 ± 2.4 days, Min: 1, Q1: 3.0, Median 4.0, Q3: 5.0, Max: 14) pain intensity was 1.81 ± 1.48 (Min: 0, Q1: 1.0, Median: 1.0; Q3: 3.0; Max: 6), Fig. 3. During follow-up at 6 weeks, 6 months, and 1 year NRS for pain was reduced to 1.33 ± 1.72 (Median: 0), 0.57 ± 1.42 (Median: 0), and 0.35 ± 1.04 (Median:0), respectively, Fig. 4. 1year following IPOM, pain at rest and in burden was reported in 2/74 (2.7 %) and 9/74 (12.2 %), respectively.

**Fig. 5:**
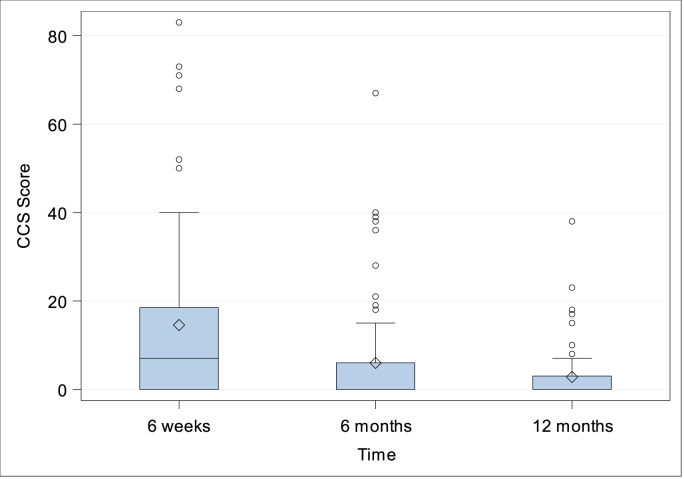
Patient-reported health-adapted quality of life measured by CCS: Total CCS Score at 6 weeks, 6 months, and 1 year follow-up.

**Fig. 6:**
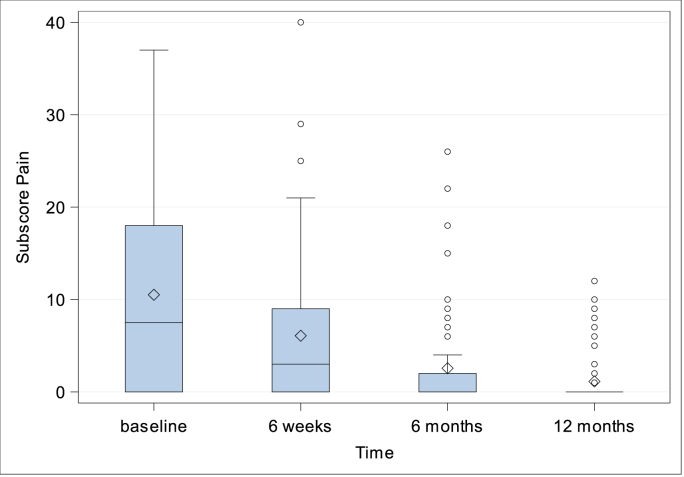
Patient-reported health-adapted quality of life measured by CCS: Subscore pain at baseline and at 6 weeks, 6 months and 1 year follow-up.

**Fig. 7:**
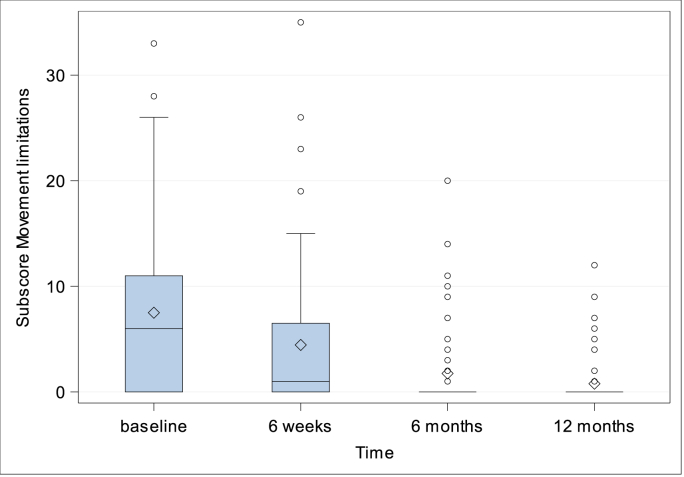
Patient-reported health-adapted quality of life measured by CCS: Subscore movement limitations at baseline and at 6 weeks, 6 months and 1 year follow-up.

**Fig. 8:**
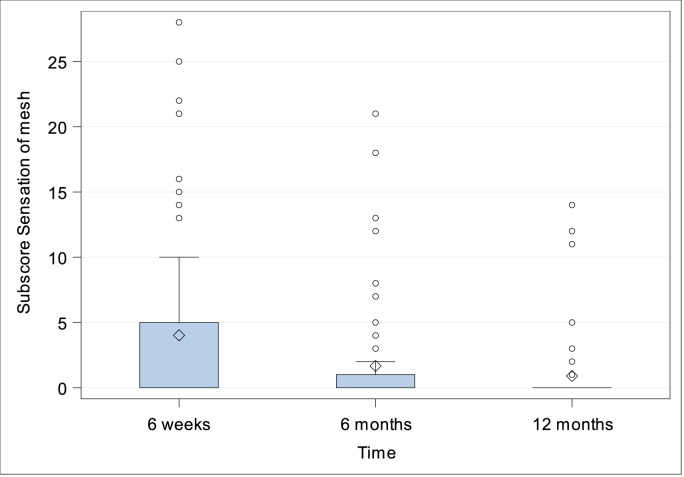
Patient-reported health-adapted quality of life measured by CCS: Subscore sensation of mesh at 6 weeks, 6 months and 1 year follow-up.

**Fig. 9:**
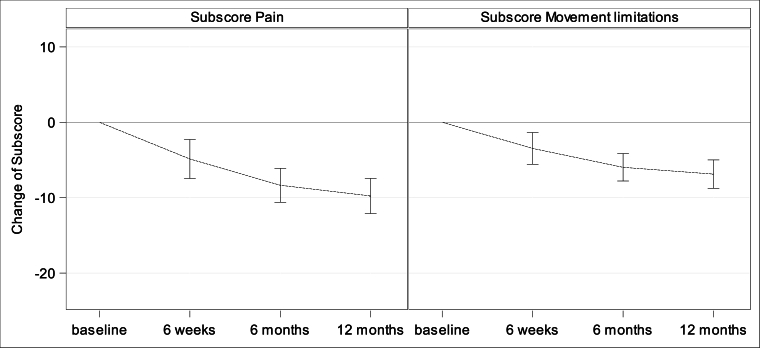
Patient-reported health-adapted quality of life measured by CCS: Mean changes of subscores pain and movement limitation during follow-up after IPOM compared to preoperative assessment.

***Quality-of-life.*** CCS Score and subscores were evaluated preoperatively (with the exception of CCS Score and subscore mesh sensation), after 6 weeks, 6 months and 1 year. The CCS total scores were 14.54 ± 19.27 (N = 76; Min: 0, Q1: 0, Median: 7.0; Q3: 18.5; Max: 83.0) after 6 weeks, 5.97 ± 12.22 (N = 75; Min: 0, Q1: 0, Median: 0; Q3: 6.0; Max: 67.0) after 6 months and 2.80 ± 6.47 (N = 71; Min: 0, Q1: 0, Median: 0; Q3: 3.0; Max: 38.0) after 1 year, Fig. 5. Subscore pain was preoperatively 10.51 ± 10.05 (N = 82; Min: 0, Q1: 0, Median: 7.5; Q3: 18.0; Max: 37.0), 6.08 ± 8.15 (N = 76; Min: 0, Q1: 0, Median: 3.0; Q3: 9.0; Max: 40.0) after 6 weeks, 2.56 ± 5.25 (N = 75; Min: 0, Q1: 0, Median: 0; Q3: 2.0; Max: 26.0) after 6 months, and 1.13 ± 2.58 (N = 71; Min: 0, Q1: 0, Median: 0; Q3: 0; Max: 12.0) after 1 year, Fig. 6. Subscore movement limitation was 7.50 ± 7.96 (N = 82; Min: 0, Q1: 0, Median: 6.0; Q3: 11.0; Max: 33.0) preoperatively, 4.45 ± 7 (N = 76; Min: 0, Q1: 0, Median: 1.0; Q3: 6.5; Max: 35.0) after 6 weeks, 1.75 ± 4.09 (N = 75; Min: 0, Q1: 0, Median: 0; Q3: 0; Max: 20.0) after 6 months, and 0.77 ± 2.22 (N = 71; Min: 0, Q1: 0, Median: 0; Q3: 0; Max: 12.0) after 1 year, Fig. 7. Subscore sensation of mesh was 4.01 ± 6.76 (N = 76 Min: 0, Q1: 0, Median: 0; Q3: 5.0; Max: 28.0) after 6 weeks, 1.67 ± 3.99 (N = 75; Min: 0, Q1: 0, Median: 0; Q3: 1.0; Max: 21.0) after 6 months, and 0.90 ± 2.69 (N = 71; Min: 0, Q1: 0, Median: 0; Q3: 0; Max: 14.0) after 1 year, Fig. 8. Subscores pain and movement limitation were both significantly reduced after 6 weeks, 6 months and 1 year compared to preoperative assessment (p < 0.001), Fig. 9.

## 4 Discussion

At the time, the LIPOM trial was planned, most available data on the Physiomesh™ and the Securestrap™ were based on industry conducted or sponsored studies. The Physiomesh™ was shown to have adequate tissue fixation and excellent tissue integration at 28, 56, and 91 days after mesh implantation in a preclinical porcine model [33]. In a randomized preclinical comparator study using a rabbit sidewall model, Physiomesh™ was demonstrated to be superior to other composite meshes regarding intraperitoneal adhesion formation [34]. In specimens of abdominal wall with ingrown Physiomesh™ harvested from rabbits 12 weeks after mesh implantation, a high mesh-tissue compliance during bi- and triaxial stress mimicking anisotropic compliance of the abdominal wall, a low Vandendael adhesion score, and mildest foreign body reaction were shown as compared to other composite meshes [24]. In 2011 Deeken and colleagues concluded in a review on available meshes for intraperitoneal use, that there are only limited data on antiadhesive properties with Physiomesh™ and that clinical trials are needed to more appropriately define the clinical effectiveness of the used antiadhesive barriers of these meshes [35]. The acute holding strength of Securestrap™ at various deployment angles was tested in a fresh porcine flank model and was shown to be comparable to other available fixation devices at 90° and to be superior in lower deployment angels [36].

These preclinical findings were supported in a single arm multicenter observational study by the International Hernia Mesh Registry, which is funded by Ethicon. Preliminary follow-up data at 1 year in 100 patients (29 incisional hernias, 71 ventral hernias) undergoing IPOM with Physiomesh™ with a follow up of 74 % or greater revealed 1 recurrence in the ventral hernia cohort, in both groups no perioperative complications and a favourable long term QoL as determined by CCS [22]. The authors concluded, that in the entire patient population the abdominal-wall-like compliance of Physiomesh™ favourably impacts hernia repair results without increase in failure or re-operative rate. In addition, Cavallaro et al. showed in 40 consecutive patients undergoing laparoscopic IHR the Securestrap™ to be safe with no increase of the risk of mesh dislocation as compared to the use of nonabsorbable tacks [37].

These data are in line with the results in the LIPOM trial, which was designed to standardize in particular surgical technique for IPOM in elective IH, that essentially encompass mesh overlap of 5 cm, mesh fixation with absorbable tacks in double crown technique using the Securestrap™ and nonabsorbable TFS at the edges of the mesh along with the application of Physiomesh™ in order to evaluate the impact on clinical and patient reported outcome and to improve comparability of data. We found a cumulative recurrence rate at 1 year of 4.1 % (95 %-CI: 0.9–11.7 %) with a Kaplan-Meier estimate for freedom-of-recurrence of 95.9 % (95 %-CI: 87.9–98.7 %). This is, subject to the described inhomogeneity of data, similar to recurrence rates around 4.7 % at 1 year given in the literature [38]. The perioperative complication rate was low with 3 superficial surgical site infections (n = 3/74 (4.1 %); 95 %-CI: 0.9–11.7 %, Clavien-Dindo grade I), which were treated without need for reoperation and 8 subcutaneous hematomas (n = 8/75 (10,7 %); 95 %-CI: 5.3–19.9) with the need of a reoperation in 2 patients (Clavien-Dindo grade IIIb). In the literature, rates of seroma formation range from 2 to 20 % [3]. In the LIPOM trial, there were 12 seromas (type II/IV: 11/1, respectively) diagnosed with the need for surgical intervention in 1 patient (Clavien-Dindo grade IIIb) resulting in a seroma rate in the upper range of 16.2 %. Patient reported pain intensity at 1 year follow-up was below preoperative values with 0.35 ± 1.04 (Median: 0) as measured by 11-point NRS supporting the applied fixation concept in our study from this point of view. Patient reported QoL measures support this observation with a decrease of the total CCS score and a significant reduction of subscores pain and movement limitation at 6 weeks, 6 months and 1 year compared to preoperative assessment.

The presented data are confirmed by the results of a non-industry-sponsored case-control study evaluating the Ventralight ST™ intraperitoneal onlay mesh in incisional and primary ventral hernia repair (EHS W1 and W2) with other composite meshes. The Physiomesh™ was applicated in the control group in 34/86 patients (39,5 %) with a mesh overlap of at least 5 cm given in 82/86 patients (95,3 %), and fixation with absorbable U-shaped-tacks only in 86.0 % (74/86). At 1 year, the follow-up rate was 84,9 % and patient reported outcome in the control group revealed in 1 out of 73 patients a „suspected“ recurrence and in terms of postoperative complications and QoL outcomes no difference compared to the treatment group [25].

The discussed data are in contrast to the results of a prospective randomized controlled clinical trial by Pawlak et al., which was designed to compare two different mesh/fixation systems in IPOM for primary and secondary verntral hernia. The study design comprised mesh fixation with a standardized application of absorbable tacks using the double-crown technique calculating the needed tack number by computer software, which was based on the HAL2010 biomathematical model ensuring a distance between tacks ranging from 1.5 to 2 cm, and absorbable TFS (PDS size 0) in a vertical line only if necessary. The study was stopped during a planned interim analysis for safety after randomization of 25 patients due to a 20 % recurrence rate during the first 6 months with most of the recurrences diagnosed between the 3rd and 6th month after implantation in the Physiomesh™/Securestrap™ group compared to no recurrence in the Ventralight™ ST/SorbaFix™ group [39]. These data are supported by the findings from the publicly funded Danish Hernia Database and the German Herniamed Registry, which is financially supported by the medical technology industry. The data from both registries led to a voluntary market withdrawal of Physiomesh™ on May 25, 2016 [40]. In the Herniamed database of electively IPOM for IH, the 1 year follow-up rate was 62,7 % (5214 out of 8319 patients) containing data files from 1380 patients following IHR with Physiomesh™. Univariable and multivariable analysis showed, that the mesh used had an influence on recurrence, on pain on exertion, and on chronic pain requiring treatment. Multivariable analysis of recurrence pointed out, that the risk of onset of a recurrence at 1 year was significantly higher after implantation of Physiomesh™ compared to other composite meshes (p < 0.001; OR 2.570, 95 % CI 2.057, 3.210). Other variables revealed by multivariable analysis to have had a significant influence on the recurrence rate were recurrent operation, larger defect size, lateral EHS classification, obesity, and larger mesh size, but significance could not be reached for fixation (p = 0.103). However, it must be pointed out, that detailled informations regarding fixation (i.e number of tacks, double crown vs. single crown, informations concerning TFS) are lacking due to the given options for documentation in the eCRF [41]. The findings of a nationwide cohort study from the Danish Ventral Hernia Database indicate, that the reoperation rate for recurrence for incisional hernias was correlated to the type of mesh and was significantly higher for Physiomesh, Parietex Composite, Ventrale Hernia Patch, and Proceed Surgical Mesh compared to Ventralight ST Mesh [42].

One might explain the favourable findings in the LIPOM trial with the exclusion of patients with recurrent IH, defect sizes more than EHS W2, lateral hernias according to the EHS classification more than L3 and the strict application of inclusion criteria for study sites in an attempt to reduce bias due to i. e. learning curve. Furthermore, in contrast to the missing detailled informations concerning mesh fixation in the Herniamed registry and the nationwide cohort study from the Danish Ventral Hernia Database and to the data published by Pawlak et al., where mesh fixation was carried out with absorbable tacks and absorbable TFS if necessary only, we applied a standardized surgical technique with mesh fixation in double crown technique with absorbable tacks (Securestrap™) along with nonabsorbable TFS at the edges of meshes. In 2015, Vogels et al. reported at 90 days after mesh implantation in a rat model significantly less adhesion formation, a significant reduction in craniocaudal mesh length, and significantly lower incorporation strenghts for Physiomesh™ compared with 2 other composite meshes, Parietene™ and Hi-Tex *Endo*-IP™. It was concluded, that there is a delay of tissue integration, which may be adressed to the fact, that the parietal side of the mesh is also coated with a poliglecaprone-25 anti-adhesive absorbable layer. However, the authors pointed out, that besides the shown distinctions between meshes, none of them are superior in all aspects required for effective and safe incisional hernia repair [43]. Based on this experimental evidence, while one has to be aware of the limited comparability and reliabilty of available animal models in hernia research as shown by Vogels et al. [44], and the described clinical findings, it can be hypothesized, that absorbable tack fixation alone or along with the optional use of absorbable TFS might not be enough to ensure proper fixation of the Physiomesh™ in order to overcome the suspected delay in tissue integration.

The LIPOM trial is not without limitations. The planned sample size of 100 patients was empirically chosen as a population large enough to evaluate the impact of our standard protocol on clinical and patient reported outcome measures and small enough to complete the study in a reasonable time frame. Due to patient selection with inclusion of only primary incisional hernias undergoing elective surgery, the results obtained might not be extrapolated to i. e. emergency treatments. Our follow-up rate for primary endpoint at 1 year of 87,1 % is high, but it might be, that patients lost to follow-up either had very bad or very favourable outcome and therefore refused to continue or did not see the need for follow-up. Follow-up at 1 year is a commonly used time point with cumulative observation of the majority of recurrences, but it is well known, that the longer patients after IHR are followed, the greater the rate of recurrence can be. However, in the cited studies from Pawlak et al. and from the Herniamed registry, there is strong evidence for the onset of recurrences very early after implantation of Physiomesh™. Therefore, the 1 year follow-up appears to provide a more than adequate time period for recurrence to develop [18,39].

The selected study format of a non-comparative, open prospective multicenter observational cohort study in the LIPOM trial potentially may limit the ability to provide useful representative informations. Lack of a control group or any element of randomization does introduce an opportunity to suggest, that change of one or several components of the proposed standardization could provide a similar or better clinical outcome. In their recommendations for reporting outcome measures in abdominal wall repair, the Palermo Group proposed observational studies such as non-comparative cohort studies as a valuable tool, when conducted properly [18]. In our study, enrollment of patients was consecutive without any pre-selection and with registration of patient's files via a web-based database in real time before the outcome is known providing a more accurate representation of patient outcome in a real world setting. Evaluation of outcome parameter included patient reported outcome measure to avoid observer related bias. Furthermore, the study design included a binding agreed standard protocol with subject to mandatory controls to almost eliminate the risk of bias due to learning curve effects and inhomogeneity in surgical technique.

Lundh and colleagues showed in a Cochrane review, that sponsorship of drug and medical device studies by the manufacturing company leads to more favourable efficacy results (RR 1.27; 95 % CI: 1.17–1.37; 25 out of 75 published records; moderate quality evidence), similar harms results (RR 1.37; 95 % CI: 0.64–2.93; 4 out of 75 published records; very low quality evidence) and more often favourable conclusions (RR 1.34; 95 % CI 1.19–1.51; low quality evidence) compared to funding by other sources. Furthermore, reported outcomes and analyses are likely to differ in order to generate more favourable conclusions (RR 0.83; 95 % CI: 0.70–0.98). The authors suggested the existence of an industry bias that cannot be explained by standard risk of bias assessments [45]. The LIPOM trial was initiated as an investigator driven study, which was funded by Ethicon for organizational concerns with no provision of free devices and no influence on design, data management, data analysis, and publishing.

## 5 Conclusions

In conclusion, the LIPOM trial showed favourable clinical and patient reported outcomes and a low rate of recurrences at 1 year after IPOM for elective IH applying a standard protocol, which particulary includes mesh fixation with absorbable tacks (Securestrap™) in double crown technique enforced by additional nonabsorbable transfascial sutures along with the use of Physiomesh™. In view of the data leading to the market withdrawal of the Physiomesh™, one might conclude, that the described standardization may have contribute to a compensation of the suspected delay in tissue integration of the Physiomesh™ due to a more proper mesh fixation compared to absorbable tack fixation with/without absorbable TFS. This may lead to the general appraisal, that the fixation technique has to be adapted to the individual characteristics of mesh, fixation devices and the anatomic landing zone of the mesh.

## CRediT authorship contribution statement

**A. Hellinger:** Writing – review & editing, Writing – original draft, Visualization, Validation, Supervision, Resources, Project administration, Methodology, Investigation, Funding acquisition, Formal analysis, Conceptualization. **F. Wotzlaw:** Writing – review & editing, Writing – original draft, Visualization, Validation, Project administration, Investigation, Formal analysis, Data curation. **V. Fackeldey:** Writing – review & editing, Supervision, Software, Investigation, Conceptualization. **G. Pistorius:** Writing – review & editing, Supervision, Investigation, Conceptualization. **M. Zdichavsky:** Writing – review & editing, Validation, Investigation. **O. Stern:** Writing – review & editing, Investigation. **R. Jünemann:** Writing – review & editing, Visualization, Validation, Software, Methodology, Investigation, Formal analysis, Data curation, Conceptualization. **A. Buia:** Writing – review & editing, Validation, Software, Investigation, Conceptualization.

## Registration

The LIPOM trial has been registered at ClinicalTrials.gov, number NCT 02089958.

## Informed consent

Informed consent was obtained from all individual participants included in the study.

## Compliance with ethical standards

All procedures performed in this study were in accordance with the ethical standards of the institutional and/or national research committee, to the guidelines of the International Conference on harmonisation (ICH) of Good Clinical Practice (GCP) and with the 1964 Helsinki declaration and its later amendments or comparable ethical standards.

## Ethical approval

The study protocol was approved by the Ethics Committees of the Landesärztekammer Hessen, the Landesärztekammer Nordrhein, and the Landesärztekammer Schleswig before start of inclusion.

## Funding

The LIPOM trial is an investigator (A.H.) initiated study (IIS) and was partly funded by Ethicon UK, a division of Johnson&Johnson Medical Ltd. (Clinical Research Funding Grant IIS 12-103). The funding source was not involved in the initiation, the conduct, the analysis, and publication of this study. All authors had independent access to the data, vouch for the completeness and accuracy of the data and had final responsibility for the decision to submit for publication.

## Declaration of competing interest

The authors declare that they have no known competing financial interests or personal relationships that could have appeared to influence the work reported in this paper.

## Appendix

Members of the LIPOM trial Study Group/Hernia panel∗

A. Buia∗, Asklepios Klinik Langen, Langen, Germany.

A. Beulecke, KRH Klinikum Grossburgwedel, Grossburgwedel, Germany.

V. Fackeldey∗, Klinik Kitzinger Land, Kitzingen, Germany.

A. Hämmerle, Sana Klinikum Hameln, Hameln, Germany.

A. Hellinger∗ and F. Wotzlaw, Klinikum Fulda, Universitätsmedizin Marburg - Campus Fulda, Fulda, Germany.

G. Pistorius∗, Sozialstiftung Bamberg, Bamberg, Germany.

O. Stern, Asklepios Klinik Wandsbek, Hamburg, Germany.

A. Türler, Johanniter-Kliniken, Bonn, Germany.

R. Wilke, Nagold Klinikverbund-Südwest, Nagold, Germany.

M. Zachert∗, Steigerwaldklinik Burgerbach, Bamberg, Germany.

M. Zdichavsky, Filderklinik, Filderstadt-Bonlanden, Germany.
